# Diagnosis of Multisystem Inflammatory Syndrome in Children in a Resource-Limited Center

**DOI:** 10.7759/cureus.22254

**Published:** 2022-02-15

**Authors:** Nishanth Sekar, Mayurathan Pakkiyaretnam, Vaithehi Francis

**Affiliations:** 1 Faculty of Health Care Sciences, Teaching Hospital Batticaloa, Batticaloa, LKA; 2 Pathophysiology, Faculty of Health Care Sciences, Batticaloa, LKA

**Keywords:** children's, intravenous immunoglobulins (ivig), corticosteroids in covid-19, sars-cov-2 related pneumonia, mis- c

## Abstract

Multisystem inflammatory syndrome in children (MIS-C) has become a serious disease entity following the high prevalence of coronavirus disease 2019 (COVID-19) infection with the involvement of gastrointestinal organs, kidneys, heart, and lungs. When the patient presents with mucocutaneous findings such as conjunctival injection, red lips, neurocognitive symptoms, swollen hands and lymphadenopathy, it is always highly recommended to exclude multisystem inflammatory syndrome.

As it affects multiple organs, it can result in more serious consequences. The manifestations depend largely on the organ involved. Therefore, successful management partly depends on the early diagnosis. Many treatment strategies have been put forth to tackle the disorder so far.

## Introduction

Multisystem inflammatory syndrome in children (MIS-C) is a condition where different organs of the body can become inflamed including gastrointestinal organs, kidneys, heart, lungs, eye, brain, and skin. It is connected to the recent coronavirus disease 2019 (COVID-19) infection and usually occurs within two to six weeks from the onset. It is a rare clinical entity where the symptoms depend on the organs involved. It is a delayed and post-infectious complication of COVID-19 [1].

As early identification and treatment are key to complete recovery, it is important to investigate patients thoroughly to make an early diagnosis and initiate the treatment as soon as possible. Although a variety of presentations are observed, gastrointestinal symptoms and fever are very common and recent studies put it as high as 80% amongst the presentations [2].

The other common presentations of MIS-C include rash, conjunctivitis, mucous membrane involvement, sore throat, neurocognitive symptoms, respiratory symptoms, and lymphadenopathy. The common investigation findings include elevated inflammatory markers like C-reactive protein (CRP), D-dimer, erythrocyte sedimentation rate (ESR), and procalcitonin with hypoalbuminemia [1]. 

The MIS-C mimics the symptoms of various other illnesses such as Kawasaki disease, toxic shock syndrome, and hemophagocytosis lymphohistiocytosis [2].

## Case presentation

**Figure 1:**
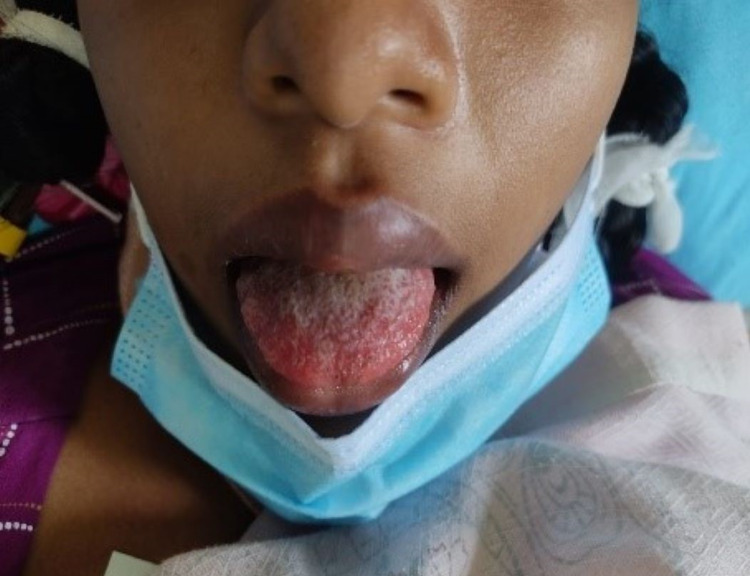
Presentation during the illness.

**Figure 2:**
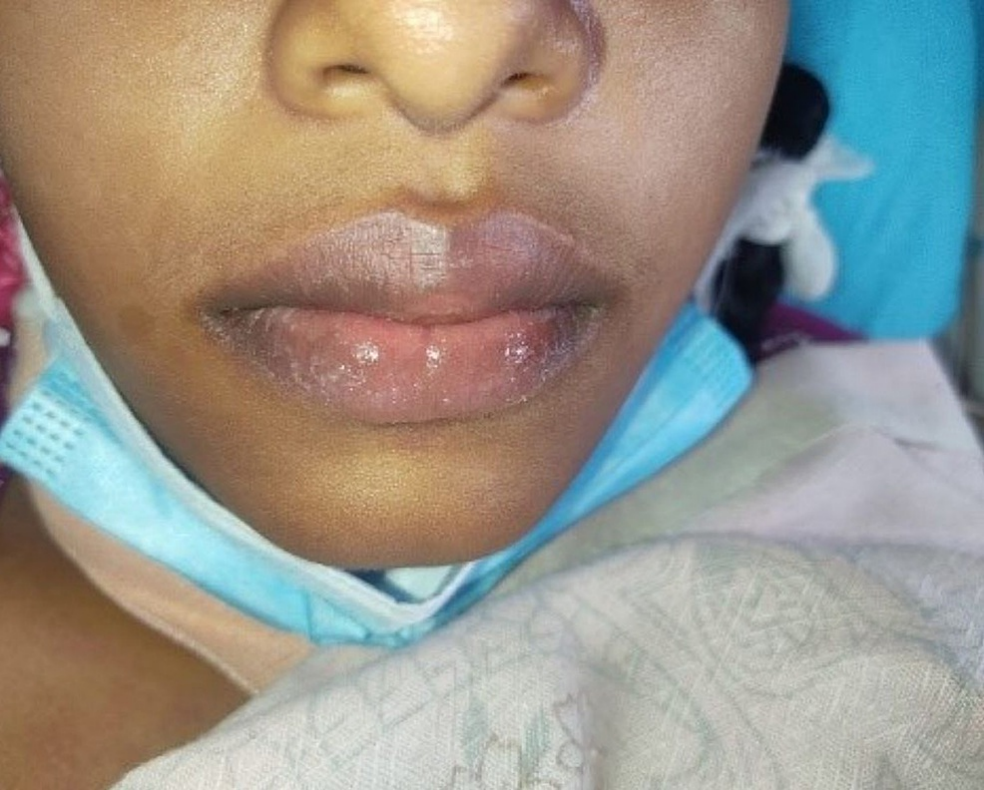
Presentation during the illness: the red lips.

**Figure 3:**
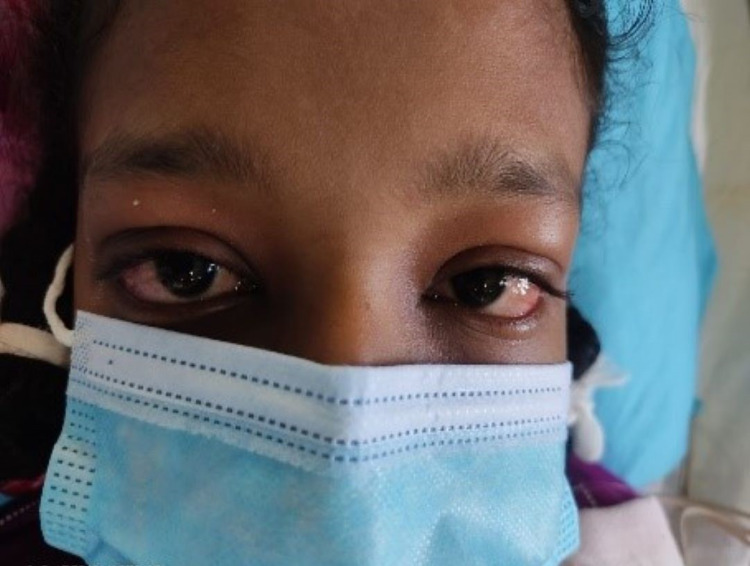
Presentation during the illness - conjunctival injection.

**Figure 4:**
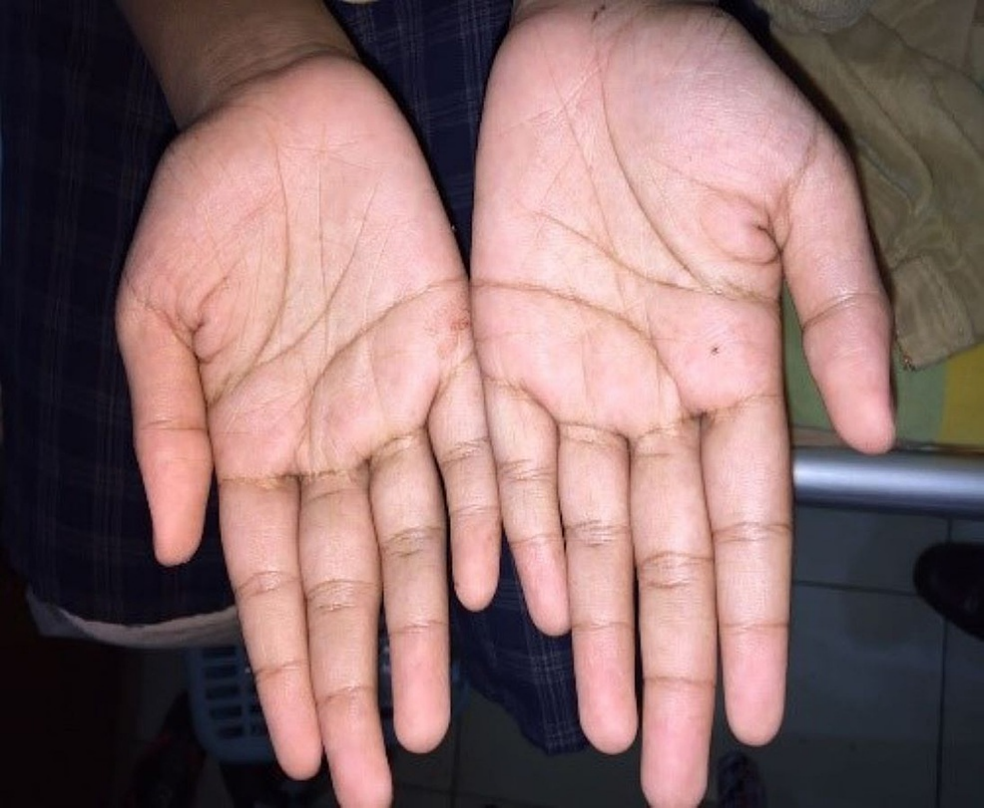
Redness of both the palms.

**Figure 5:**
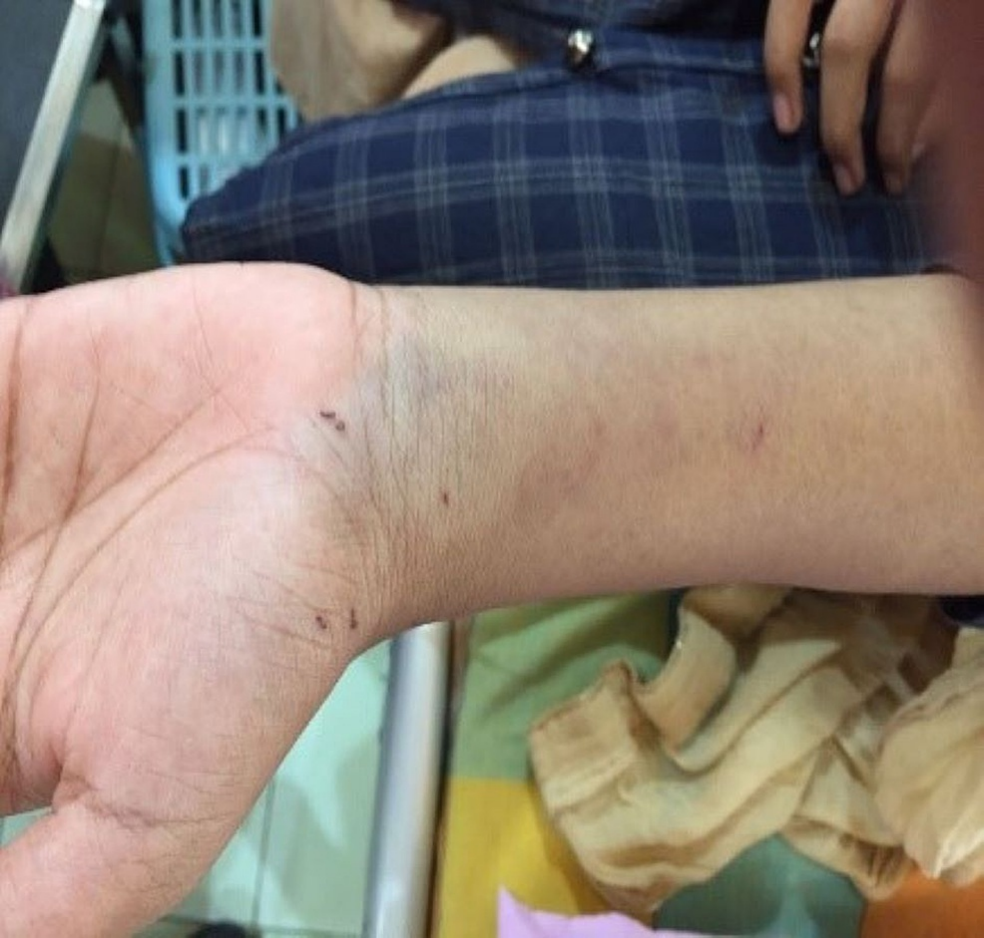
Erythematous rash on the forearm.

We report a case of a 17-year-old South Asian girl who presented with fever with nausea, vomiting, watery diarrhea, and redness in both eyes for four days duration. She vaguely remembered any respiratory symptoms within the last two to six weeks prior to the current illness. She had no urinary symptoms. Neither did she have any headache. She did not have any blood or mucoid admixture in the stool (Figures 1-5). 

On examination, she was febrile and had a bilateral conjunctival injection and maculopapular rash on the bilateral upper and lower limbs. There was no neck stiffness and Kernig's sign was negative. There was no ankle edema. She was tachycardic with a pulse rate of 165 beats/min, regular and good volume with a blood pressure of 80/52 mmHg. There was no murmur on auscultation. Respiratory examination showed a respiratory rate of 22 breaths/min and SpO2 99% on room air. Lungs were clear on auscultation with normal breath sounds. On abdominal examination, there was no hepatosplenomegaly and the neurology examination was unremarkable. The investigation summary is as follows (Table 1). 

**Table 1:** The list of investigations done. CRP, C-reactive protein; LDH, lactate dehydrogenase; ESR, erythrocyte sedimentation rate; FBC, full blood count; WBC, white blood cell count; hpf, high power field; INR, international normalized ratio; APTT, activated partial thromboplastin clotting time; ECG, electrocardiogram; LDH, lactate dehydrogenase

Investigations			
Date	26/08	27/08	28/08
FBC			
WBC (10^3^)/µL	9.42	17.5	16.6
Neutrophils	90%	89%	91.2%
Lymphocytes	3%	5.8%	5.2%
Platelets (10^3^)/µL	100	138	133
Hemoglobin (g/dL)	12	11.1	11.1
CRP (mg/L)	280	289	216
ESR (mm/h)			
LDH (U/L) <234		409	
Serum ferritin (ng/mL)		800	
Troponin I (ng/mL) <0.12		3.27	
D dimer (g/mL) <1		8.52	
Serum sodium(mmol/L)		140	141
Serum potassium (mmol/L)		3.9	4
Serum calcium (mmol/L)		2.1	
Serum creatinine (µmol/L)	88	102	74
Blood urea (mmol/L)	5.7	7.6	2.8
Alanine transaminase (U/L)	28	39	36
Aspartate transaminase (U/L)	31	64	43
Alkaline phosphatase (U/L)	63	62	59
Gamma glutamyl transferase (U/L)	65	64	60
Total protein (g/L)	65	58	71
Serum albumin (g/L)	29	29	21
Serum globulin (g/L)	36	33	52
Total bilirubin (µmol/L)	11	10.7	10
INR	1.82	2.23	1.2
APTT	23.3	34.8	31
Procalcitonin (ng/mL)			83
Urine full report pus cells and red cells and albumin (/hpf)	4.-6 & 8-10 & Trace		
Blood culture	No growth		
Urine culture	No growth		
Blood picture	Likely due to infection		
Chest X-ray	Normal		
ECG	Sinus tachycardia		
2D echocardiography		Mild global hypokinesia EF 50-55%	
Ultrasound scan abdomen		R/S pleural effusion Peri cholic fluid collection	

Amongst the clinical manifestations of MIS-C, fever for more than three days, mucocutaneous symptoms like rash, conjunctivitis, red swollen lips, and strawberry tongue along with myalgia were all present. On top of that, the patient had gastrointestinal symptoms in the form of nausea, vomiting, and diarrhea. Neurocognitive symptoms like irritability and headache were also present. 2D-Echocardiography confirmed cardiac involvement with mild global hypokinesia with an ejection fraction of 50%. Rapid antigen test and COVID-19 polymerase chain reaction (PCR) were negative. COVID-19 antibody testing revealed a past infection. 

**Figure 6:**
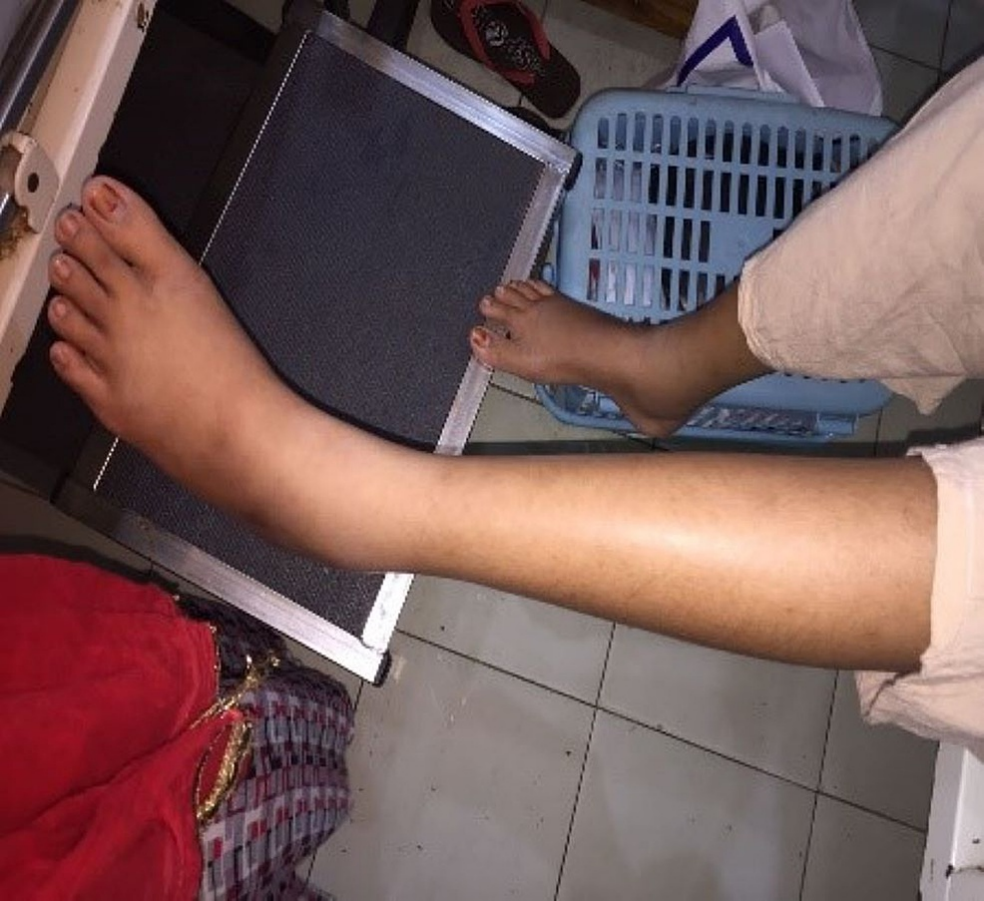
Appearance of the legs after recovery.

**Figure 7:**
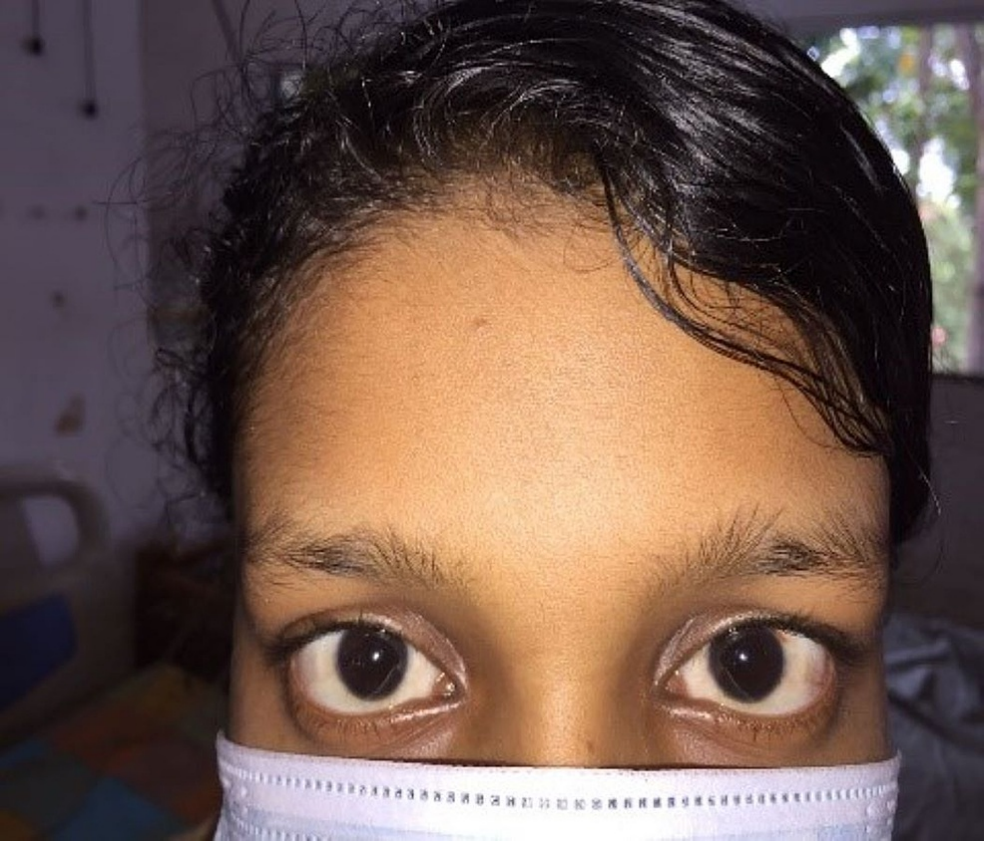
Appearance of the eyes after recovery.

**Figure 8:**
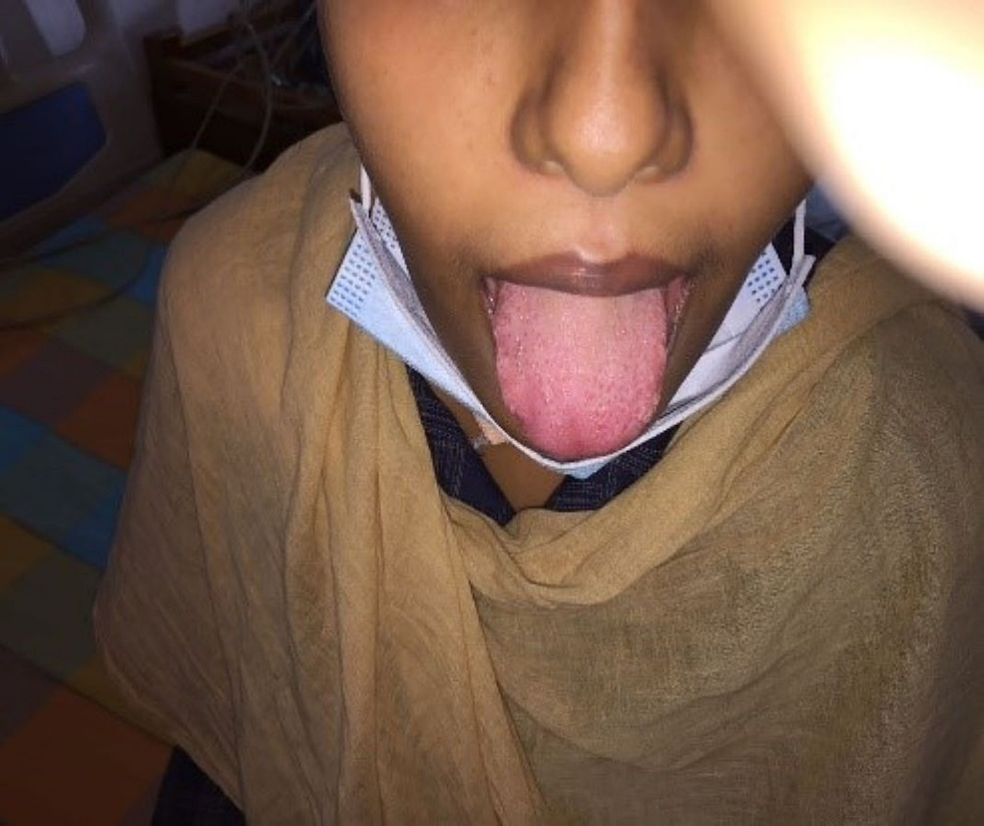
Appearance of the mouth and tongue after recovery.

She was started on intravenous immunoglobulin (IVIG) 2 g/kg as an infusion over 12 h. Aspirin 75 mg was also started. After the cultures, broad-spectrum antibiotics such as IV cefotaxime and vancomycin were initiated. Upon admission, subcutaneous enoxaparin sodium 30 mg mane was also added. IVIG was followed by IV methylprednisolone 1 g per day pulse therapy for three days. It was transitioned to oral prednisolone 1 mg/kg regimen which was planned to be tapered off over the period of four weeks. The patient made a complete recovery with the satisfactory improvement of the clinical parameters and significantly reduced inflammatory markers after five days of initiation of therapy (Figures 6-8). 

## Discussion

In patients younger than 20 years old, who present with fever for more than three days and marked gastrointestinal symptoms with evidence of past COVID-19 infection, MIS-C must be considered as a possible differential diagnosis [3]. It must be remembered that this particular syndrome must be considered in children with features of typical and atypical Kawasaki disease and toxic shock syndrome. Other probable differential diagnoses include bacterial sepsis, severe acute COVID-19 infection, systemic lupus erythematosus (SLE), hemophagocytosis lymphohistiocytosis (HLH), and macrophage activation syndrome (MAS) [4]. In our patient, we excluded the most probable differential diagnosis although some of the investigations were not available. 

According to the World Health Organization (WHO) case definition criteria, our patient had a fever for more than three days with persistent hypotension, acute gastrointestinal symptoms like diarrhea, coagulopathy (international normalized ratio, INR: 2.23), and features of cardiac dysfunction in the form of mild global hypokinesia. In addition, the biomarkers of inflammation ESR, C-reactive protein (CRP), serum ferritin, D-dimer, creatinine phosphokinase (CPK), and lactate dehydrogenase (LDH) were elevated in the absence of other obvious microbial causes of inflammation (blood culture and urine culture were all negative). And there was evidence of past COVID-19 infection (positive COVID-19 antibody testing) [2].

The main mode of therapy in the disease entity is immunosuppression [5]. Along with IVIG (2 g/kg over 8-12 h), steroids have a key role to play in the treatment of MIS- C. Since the thromboembolic complications are described, enoxaparin is also warranted. Antiplatelet mainly aspirin is continued for four weeks from the outset and sometimes until the coronary arteries become normal. In case of refractory MIS-C where the disease fails to achieve remission, a second dose of IVIG is preferred with pulse doses of IV methylprednisolone (30 mg/kg dose- maximum 1 g daily dose) for three to five days [6]. In our patient, the accomplishment of the recrudescence with the first dose of IVIG was evident so that a second dose was not warranted. 

Usage of adjunctive therapies is uncertain. IL-1 inhibitors (IV anakinra 2-10 mg/kg/dose - maximum 100 mg dose), IL-6 inhibitors (tocilizumab 4-8 mg/kg per dose) [7], and convalescent plasma from recovered COVID-19 patients are tried.

The follow-up plan includes monitoring inflammatory markers [erythrocyte sedimentation rate (ESR) and serum ferritin] and echocardiography which is done after one to two weeks in those who have normal function and normal coronary artery dimensions and after two to three days in those with coronary artery dilation until it is stable in size. 

In our patient, follow-up 2D echocardiography done in two weeks showed normal cardiac function with an ejection fraction of 60%. 

There are several cases of MIS-C reported all around the world. In Moroccan children, there were reported cases of the multisystem inflammatory syndrome as a part of severe severe acute respiratory syndrome coronavirus 2 (SARS-CoV2) infection [8]. In Sri Lanka, only a few are reported and are successfully treated.

## Conclusions

In the current COVID-19 prevalent situation, MIS-C must be one of the most important differential diagnoses when a patient presents with mucocutaneous manifestations and multiorgan involvement with any evidence of past COVID-19 infection. Prognosis is excellent following appropriate and early aggressive treatment. Therefore, it is worthwhile to initiate immunosuppressive therapy as early as possible considering the devastating nature of the illness. 

## References

[1] (2021). COVID-19: multisystem inflammatory syndrome in children (MIS-C) management and outcome. https://www.uptodate.com/contents/covid-19-multisystem-inflammatory-syndrome-in-children-mis-c-clinical-features-evaluation-and-diagnosis.

[2] (2021). Multisystem inflammatory syndrome in children and adolescents temporally related to COVID-19. https://www.who.int/news-room/commentaries/detail/multisystem-inflammatory-syndrome-in-children-and-adolescents-with-covid-19.

[3] Haoudar A, Chekhlabi N, Eljazouly M (2021). Severe SARS-CoV-2 infection: a multisystem inflammatory syndrome in Moroccan children. Cureus.

[4] (2021). Information for healthcare providers about multisystem inflammatory syndrome in children (MIS-C). https://www.cdc.gov/mis/mis-c/hcp/index.html.

[5] Matsuda EM, Santos SA, Castejon MJ, Ahagon CM, Campos IB, Brígido LF (2020). COVID-19 in children: a case report of multisystem inflammatory syndrome (MIS-C) in São Paulo, Brazil. Braz J Infect Dis.

[6] Pegoraro F, Trapani S, Indolfi G (2021). Gastrointestinal, hepatic and pancreatic manifestations of COVID-19 in children. Clin Res Hepatol Gastroenterol.

[7] (2021). Multisystem inflammatory syndrome in children: a manifestation of COVID-19. https://consultqd.clevelandclinic.org/multisystem-inflammatory-syndrome-in-children-an-emerging-manifestation-of-covid-19/.

[8] (2022). What is the role of interleukin (IL) inhibitors in the treatment of coronavirus disease 2019 (COVID-19)?. https://www.medscape.com/answers/2500114-197455/what-is-the-role-of-interleukin-il-inhibitors-in-the-treatment-of-coronavirus-disease-2019-covid-19#:~:text=Interleukin%20(IL)%20inhibitors%20may%20ameliorate,TNF%CE%B1)%20and%20other%20inflammatory%20mediators..

